# Death is common, so is understanding it: the concept of death in other species

**DOI:** 10.1007/s11229-020-02882-y

**Published:** 2020-09-29

**Authors:** Susana Monsó, Antonio J. Osuna-Mascaró

**Affiliations:** 1 Unit of Ethics and Human-Animal Studies, Messerli Research Institute, University of Veterinary Medicine, Vienna, Austria; 2 Unit of Comparative Cognition, Messerli Research Institute, University of Veterinary Medicine, Vienna, Austria

**Keywords:** Comparative thanatology, Nonhuman animals, Concept of death, Grief, Anthropocentrism, Predation, Thanatosis

## Abstract

Comparative thanatologists study the responses to the dead and the dying in nonhuman animals. Despite the wide variety of thanatological behaviours that have been documented in several different species, comparative thanatologists assume that the concept of death (CoD) is very difficult to acquire and will be a rare cognitive feat once we move past the human species. In this paper, we argue that this assumption is based on two forms of anthropocentrism: (1) an intellectual anthropocentrism, which leads to an over-intellectualisation of the CoD, and (2) an emotional anthropocentrism, which yields an excessive focus on grief as a reaction to death. Contrary to what these two forms of anthropocentrism suggest, we argue that the CoD requires relatively little cognitive complexity and that it can emerge independently from mourning behaviour. Moreover, if we turn towards the natural world, we can see that the minimal cognitive requirements for a CoD are in fact met by many nonhuman species and there are multiple learning pathways and opportunities for animals in the wild to develop a CoD. This allows us to conclude that the CoD will be relatively easy to acquire and, so, we can expect it to be fairly common in nature.

## Introduction

Comparative thanatologists attempt to uncover the proximate mechanisms involved in the responses to the dead and the dying across animal^1^ species, as well as the ultimate functions behind these mechanisms. This area of study is filled with difficulties, given that ethical constraints make the use of experiments particularly tricky (Gonçalves and Biro 2018; Monsó 2019), and so scientists must rely more than usual on opportunistic observations gathered in the wild. Despite these methodological hurdles, comparative thanatologists tend to agree on the idea that the concept of death (hereafter, ‘CoD’) is an unusual cognitive feat once we move beyond the human species. For instance, despite the growing number of thanatological reports gathered on monkeys, who show huge variability in their reactions to death, De Marco et al. assert: “there is no reason to believe that monkeys have a concept of death” (De Marco et al. 2018, p. 58). Similarly, after a detailed review of the evidence, Gonçalves and Carvalho state that great apes —our closest living relatives—are the “likeliest candidates for achieving aspects of a human‐like concept of death,” but at the same time “the burden of proof still awaits future research” (Gonçalves and Carvalho 2019, p. 1519).

Underlying this tacit agreement is the assumption that a CoD is very difficult to acquire, and that only species or individuals with high cognitive sophistication (such as great apes) can be capable of acquiring it. In this paper, we are going to argue against this assumption. We will do this in two steps. First, in Sect. 2, we will show that this presupposition stems from two forms of anthropocentrism that underlie scientists’ reflections on death: (1) an intellectual anthropocentrism, which leads to excessively demanding accounts of the CoD, and (2) an emotional anthropocentrism, which yields an unwarranted focus on grief as a reaction to death. Contrary to what these two forms of anthropocentrism suggest, we will argue that the CoD requires relatively little cognitive complexity and that it can emerge independently of mourning behaviour. Second, in Sect. 3, we will defend that a wide range of species and situations can allow for a CoD to arise in nature. To be clear, we do not intend to take a concrete stand on exactly *which* species can possess a CoD, since that is an empirical matter that is beyond the scope of this paper. Instead, we will examine the cognitive requirements of the CoD and show how, coupling this analysis with biological and ecological considerations, we can predict that the CoD is relatively easy to acquire and consequently much more prevalent in nature than is usually presupposed.

## Anthropocentrism in comparative thanatology

Comparative thanatologists^2^ have uncovered two main ways in which animals respond to death. On the one hand, there are some behaviours triggered by corpses that are clearly shaped by natural selection, rigid, and homogeneous along the individuals of a single species. These reactions to death seem to exist in a wide variety of organisms, with the most distinctive example represented by the stereotypical responses of eusocial insects. These are triggered by certain chemical characteristics of corpses, and are usually related to hygienic or prophylactic needs (Sun and Zhou 2013). Necrophoresis (the removal of dead conspecifics from the nest/surroundings) and necrophobia (the avoidance of dead hetero- and conspecifics) are two typical examples of behavioural responses that are triggered solely by the perception of death and are not dependent on learning (Gonçalves and Biro 2018). On the other hand, comparative thanatologists have also documented, in several avian and mammalian species, responses to death that are more flexible, vary within a species, and lack a clear adaptive value. These range from affiliative behaviours, like prolonged carrying, grooming, or nurturing corpses, to aggressive, exploratory, cannibalistic, and sexual behaviours. The fact that so many different kinds of responses can be triggered by one and the same stimulus points to these behaviours being mediated by cognitive mechanisms (Allen 1999).^3^

When discussing whether animals can acquire a CoD, we are interested in the mechanisms underlying this second class of behaviours. The question is whether, through non-stereotypical interactions with corpses, animals can come to acquire an understanding of what it means to be dead. As we saw in the introduction, a common assumption among comparative thanatologists is that the CoD is very difficult to acquire and only within the reach of some individuals of cognitively sophisticated species. In this section, we want to show how this assumption stems from two unwarranted forms of anthropocentrism, which we call intellectual anthropocentrism and emotional anthropocentrism. The first one amounts to the assumption that the only way of *understanding* death is the human way; the second one is the idea that the only way of *emotionally reacting* to death is the human way. These two forms of anthropocentrism have led to a distorted perception of how prevalent the CoD is likely to be in nature.

### Intellectual anthropocentrism

Comparative thanatologists are aware that their topic of study comes with the potential threat of anthropomorphism, and often emphasise the need to protect their science from this danger (e.g. Brosnan and Vonk 2019; Das et al. 2019). However, they also tend to work under the anthropocentric^4^ assumption that the only possible way of thinking about death is the human way, so that animals either possess *our* CoD or none at all.^5^ As we will show in this subsection, this results in a tendency to over-intellectualise what it means to understand death. We call this intellectual anthropocentrism. Against it, we will defend Monsó’s (2019) notion of a *minimal* CoD, and show how it can account for what it means to understand death at the most fundamental level while also leaving space for the ways in which different species and individuals might understand death. The defence of this minimal CoD will illustrate how many demanding capacities that have been linked to the CoD can in fact be relinquished as necessary conditions for a minimal understanding^6^ of death.

One way in which intellectual anthropocentrism manifests itself is through the depiction of the CoD as an *abstract* concept. Brosnan and Vonk, for instance, argue:

Unobservables are hypothetical constructs that cannot, in principle, assume physical form and cannot be directly perceived (Vonk and Povinelli 2006). Death is one such construct. Although we can observe the process of dying and the physical remains of the deceased individual, we cannot perceive death itself. (Brosnan and Vonk 2019, p. 79).

Brosnan and Vonk use the distinction between, on the one hand, the process of dying and the resulting state of being dead and, on the other hand, death itself, which they consider to be a hypothetical construct, to argue that only animals who can reason about unobservables can acquire a CoD. However, death is only an abstract concept when one has the human perspective in mind. When we speak of humanity’s fear of death, for instance, we are thinking of death in abstract terms, as something that we know will inevitably befall all of us, but which we cannot point to or perceive with any of our senses. Depictions of death as a hooded figure with a scythe are attempts to make concrete this unobservable entity that haunts our lives. While death in this sense is ‘unobservable,’ we disagree with Brosnan and Vonk’s claim that this warrants us considering death as a hypothetical construct. The process of dying and the state of being dead are both very concrete and perceptually accessible entities. Once they occur they are neither ‘hypothetical’ nor ‘constructed.’ Pace Brosnan and Vonk, death can and does assume a physical form whenever it happens. The hypothetical and constructed nature of death only applies to it as our inevitable and not-yet-fulfilled destiny. However, it is unwarranted to assume, without further argument, that this is what we are talking about when discussing whether animals can understand death. It amounts to departing from one of the most sophisticated notions of death and asking whether animals can have that CoD, i.e., *our* CoD.^7^ Understood like this, the question becomes uninteresting: it is self-evident that creatures without a linguistic capacity that can enable an oral culture of narratives surrounding death cannot reach as sophisticated a notion of death as ours.

We believe that the interesting question, understood as the one that leaves room for discussion, is not whether animals are capable of developing a CoD that is as complex as our own, but whether they can develop anything that counts as a CoD at all. This means that our point of departure should be the *minimally sufficient* conditions for understanding death. Only when we have established that animals can reach a minimal understanding of death should we inquire into the level of sophistication that this understanding can reach. This makes more sense methodologically speaking, since it reduces the risk of false negatives. At the same time, the question should be posed in a way that doesn’t stack the cards in favour of human uniqueness. This means approaching it in a way that can accommodate the ecology of different species and doesn’t exclude non-linguistic animals a priori from the class of beings who can think about death. A balance should thus be reached between developing an account of the CoD that allows for inter- and intra-specific variation and one that enables us to meaningfully attribute an understanding of death to the species who possess it.

Monsó (2019) developed a minimal account of the CoD that is meant to accommodate these requirements. She defined this concept as follows:

A creature can be credited with a minimal concept of death once she classifies some dead individuals as dead with some reliability, where ‘dead’ is understood as a property that pertains to beings who:

- are expected to have the cluster of functions characteristic of living beings, but.
- lack the cluster of functions characteristic of living beings, and.
- cannot recover the cluster of functions characteristic of living beings. (p. 9).

This definition is meant to provide necessary and sufficient conditions to be credited with a CoD.^8^ However, the phrase “the cluster of functions characteristic of living beings” is purposefully vague in order to allow for inter- and intra-specific variability, since different species and individuals can have different notions of what characterises living beings, and consequently different CoDs (p. 10). This definition can also accommodate non-linguistic thinking. The three conditions (a)–(c) can all be reached through sensory perception and don’t require the animals reasoning about unobservable entities. Condition (a) is reached through an accumulation of experiences with beings of a certain kind, which results in the development of an expectation regarding how they typically behave. Condition (b), in turn, results from the violation of an expectation upon encountering a being who is not exhibiting these characteristic behaviours. And lastly, condition (c) emerges from an accumulation of past encounters with beings in condition (b), which enables learning that the state cannot be reversed. At the same time, there is no reason to think that this definition requires analogical reasoning or any other form of higher-order cognition (contrary to what is proposed by e.g. Gonçalves and Carvalho 2019), since all this CoD allows is to process what has happened to an individual who has died, and does not, on its own, enable any predictions regarding what might happen in the future to oneself and others who are currently alive.

Monsó reaches this definition through an analysis of the seven sub-components of the CoD that developmental psychologists use to determine how children understand death at different developmental stages. These seven sub-components are: (1) *non-functionality* (death stops all bodily and mental functions); (2) *irreversibility* (death is a permanent state); (3) *universality* (death affects all and only living beings); (4) *personal mortality* (we ourselves will also die); (5) *inevitability* (death cannot be postponed forever); (6) *causality* (death is linked to certain causes); and (7) *unpredictability* (the exact timing of death cannot be foreseen) (Monsó 2019, Sect. 3.1; see also Slaughter 2005). According to Monsó’s analysis, only the first two sub-components are necessary for a minimal understanding of death. At its very minimum, death is the irreversible cessation of the functions characteristic of living beings (of that sort). If we grant that we only need non-functionality and irreversibility for a minimal CoD, this allows us to sidestep the idea that death is an abstract concept, because these two sub-components (unlike inevitability, unpredictability, and, to a certain extent, personal mortality) can be grasped on the basis of perceptual experiences and don’t require a capacity to reason about unobservables.

Several comparative thanatologists have granted that some animal species can probably process non-functionality and irreversibility (Anderson 2018; Das et al. 2019; Gonçalves and Carvalho 2019; Li et al. 2012; Masi 2020; Watts 2020). However, they do not consider this enough to establish that animals can have a CoD because they do not operate with the idea of a minimal CoD. Instead, they try to determine whether animals have a *human-like* CoD, so they point to the absence of the other sub-components to substantiate their claim that animals have at best only an incomplete CoD. We have already argued that this intellectual anthropocentric approach amounts to a red herring, but to avoid begging the question in favour of Monsó’s minimal CoD, it is worth briefly considering whether the other sub-components could be considered necessary for a CoD.

Some texts mention universality and causality as two basic sub-components in addition to non-functionality and irreversibility (Anderson 2018; Gonçalves and Biro 2018; Gonçalves and Carvalho 2019). However, no real arguments are given in defence of this view, which appears to be simply inherited from thanatological studies in developmental psychology. If we were to incorporate universality and causality as necessary sub-components, these could not be understood in their full complexity. A complete comprehension of the universality of death would require grouping all living beings that an animal can perceive and interact with (i.e. con- and heterospecific animals, as well as plants and fungi) under the same category as ‘things that can die.’ This seems unreasonable, insofar as animals are simply unlikely to *care* about whether all of these beings are capable of falling under the extension of the CoD (Allen and Hauser 1991, p. 237), and moreover, even for humans it is difficult to establish precisely which beings fall under this extension, given the biological debates surrounding the notion of life. Similarly, a precise mechanistic understanding of the causality behind death is something that, even among humans, is probably restricted to a few experts such as pathologists, so it is irrational to expect it from animals who don’t have access to the requisite body of knowledge.

If, on the contrary, we understand causality and universality in minimal terms, as the capacities to associate death with certain causes (e.g. associating Jones’ irreversible loss of functionality with her encounter with a leopard) and to make inductive generalisations about death (e.g. realising that what happened to Jones could happen to anyone else who encounters a leopard), this could be within the reach of many nonhuman species. It is thus possible that the *natural* CoD (as opposed to the *minimal* CoD) often incorporates these two sub-components.^9^ However, we follow Monsó (2019) in thinking that understanding death in *minimal* terms does not require either of them. One can grasp what has happened to an individual who died without knowing that this can happen to other living beings and without being able to attribute it to concrete causes. One can believe that death is something that happens *randomly* to *some* individuals and still be able to process the death of an animal correctly, in the sense of understanding that this individual will no longer be able to do the sorts of things that living individuals of her kind typically do and that this is a permanent state.

What about the other three sub-components? Unpredictability is not mentioned in the comparative thanatology literature, possibly because, as Monsó (2019) notes, death is not inherently unpredictable. The exact timing of natural death cannot be predicted with certainty, but we can make approximate guesses based on, for instance, the average lifespan of a species. And the timing of death in other contexts, such as predation, can be predicted with higher precision. In contrast to unpredictability, the other two sub-components, inevitability and personal mortality, are occasionally mentioned and they contribute to the over-intellectualisation of the CoD. Monsó (2019) argues that neither of these two components are necessary for a minimal CoD on the grounds that, if we can relinquish universality, we can let go of inevitability and personal mortality, since the latter are entailed by the former. However, as we mentioned in the previous paragraph, if universality is present in nonhuman CoDs, it will likely take the form of the ability to perform inductive generalisations about death. This, at best, can yield the belief that all individuals *can* die, which is different from the idea that all individuals *will* die (Anderson 2018). It is the latter belief, and not the former, which is linked to inevitability and to personal mortality understood as something inescapable.

It seems unlikely that non-linguistic animals can develop a notion of the inevitability of their own and others’ death, but comparative thanatologists sometimes bring this up in their discussions of what it would take for animals to understand death (e.g. Anderson 2018; Brosnan and Vonk 2019). We believe that this has to do with the strong meaning that humans attach to death, which is precisely linked to its being the unavoidable fate of ourselves and those we love. This is what makes death so terrifying, and it can have such a strong influence on our lives that there is even a psychological discipline—terror management theory—devoted entirely to how humans cope with this fear (Greenberg and Arndt 2012).^10^ But this gives us a reason to think that, if animals can develop a CoD, it is more likely *not* to have these two components. This has to do with the evolutionary forces that could drive the emergence of a CoD. Gonçalves and Carvalho point to some evolutionary advantages that could come from interacting with corpses: it could “(i) promote more rapid re‐categorisation from living to dead; (ii) decrease costly vigilance/caregiving behaviours; (iii) [be] crucial to the management of grieving responses; (iv) update individual position in the group hierarchy; and (v) accelerate the formation of new social bonds” (Gonçalves and Carvalho 2019, p. 1521). Reaping all of these benefits only requires the animal to develop a minimal CoD. In contrast, it has been argued that selective pressures are unlikely to have pushed for animals to learn about the inevitability of death. According to this view, defended, for instance, by Varki (2009), natural selection is likely to have acted *against* it, given the fitness-diminishing fear that would come from the knowledge of the inevitability of one’s death.

An animal who cannot grasp inevitability and personal mortality will be missing a sense of the tragedy of mortal life. In this sense, we can expect certain emotions or anxieties to be absent in her mental world. However, this does not seem sufficient to claim that she lacks a CoD, if she is able to correctly process what the deaths of others mean. Moreover, following Monsó (2019), we can note that if science were to advance sufficiently to grant us a way of postponing death forever, this would not fundamentally alter our notion of death. Although our lives would be lived differently, and the accidental death of someone we love might be even more tragic, dying in this world would still mean the same thing: an irreversible cessation of an individual’s living functions. Death would still mean this even if we knew that it could be avoided or that it won’t apply to ourselves. Therefore, inevitability and personal mortality can be relinquished in a minimal CoD.

Even if we concede that death in minimal terms is just irreversible non-functionality, this does not automatically free us from intellectual anthropocentrism. An example of this is provided by Brosnan and Vonk, who describe how one would come to grasp irreversible non-functionality as follows:

To properly represent death, an individual must comprehend the finality that comes with it. It is possible to imagine a number of constructs that together comprise the capacity to conceive of death. For example, first one must appreciate that physical objects—including living beings—continue in time and space; that is, they continue to exist when they are not observable (i.e., object permanence). Conversely, one must appreciate that objects can also cease to exist. A first test of this construct is to examine whether nonhumans can appreciate an absence of objects for a given category. This notion can then be extended to incorporate the idea that objects once present are now absent. To imagine death, one must then understand that this absence is permanent at some point. Lastly, one must generalize this idea of the disappearance of objects to understand the cessation of life in a living being. (Brosnan and Vonk 2019, p. 80).

We believe that Brosnan and Vonk are mistaken. There is, firstly, no reason to believe that the CoD must necessarily be linked to animals’ physical cognition nor to their notion of object permanence. As Gonçalves and Biro (2018) note, it is likely that animals categorise animate and inanimate entities as two different things and that they process them with two different cognitive systems. But more importantly, Brosnan and Vonk seem to be once again departing from a human, and more concretely, a WEIRD^11^ perspective. It is the contingent ways in which WEIRD humans relate to death that make it appear primarily as an absence. When a loved one dies, their body is taken from our sight, and we have no way of confronting their death but through this absence. For an animal in the wild, death need not equate an absence. It might instead mean, for instance, a full belly. But crucially, placing too much emphasis on the idiosyncrasies of the human, WEIRD relationship with death detracts from the fact that corpses are ultimately just broken, irreparable bodies. Grasping this does not require a concept of absence. An implicit expectation regarding the typical behaviours of living beings of a certain kind will do the job. Once this expectation is broken and becomes explicit, and an animal can process that this particular being will not be exhibiting those functions anymore, then that animal has acquired a minimal CoD.

The anthropocentric tendency to over-intellectualise the CoD is mirrored in the requirement, present in Brosnan and Vonk (2019) and Gonçalves and Carvalho (2019), that a CoD incorporate a theory of mind, or the ability to attribute mental states to others. This is because, in the traditional understanding of non-functionality, this is understood as the cessation of all bodily and *mental* functions (Slaughter 2005). Given that the attribution of a theory of mind to animals seems to rest on shaky grounds, even in the case of chimpanzees (see Andrews 2017), this is seen as a reason against the possibility that animals may have a CoD. However, it only makes sense to require that a CoD incorporate an understanding of the cessation of mental functions in those cases in which an animal indeed possesses a theory of mind. As noted by Monsó (2019), if an animal develops a notion of non-functionality, this will necessarily mirror her notion of functionality. That it is unreasonable to expect otherwise can be illustrated with a simple thought experiment: imagine that scientists found out about a certain physiological process that characterises the human living body, let’s call it X. Suppose that we knew nothing about X until the moment it was discovered. Clearly it would not make sense to say that until we learnt about X we had no CoD simply because we didn’t know that with death came the cessation of X. We believe that, for those animals who lack a theory of mind, the mental states of others are equivalent to this X. Thus, it is absurd to claim that they can’t have a CoD unless they have a theory of mind. Any CoD will incorporate the understanding that the individual in question has of what characterises living beings (of that sort), with whatever limitations this understanding comes with.

As our analysis shows, if we abandon the intellectual anthropocentric stance, the CoD need not be understood in cognitively demanding terms. At its very basic, understanding death means first expecting a dead individual to exhibit her characteristic living functions, and then grasping her irreversible non-functionality. This can be reached through first-order thinking, so there is no need to conceive the CoD as an abstract notion. The sub-components universality, causality, unpredictability, inevitability, and personal mortality can all be relinquished in a minimal CoD. And lastly, a complex understanding of the functionality of living beings, including e.g. a theory of mind, is not necessary for a basic understanding of death.

### Emotional anthropocentrism

In the previous section, we argued that there is a tendency among comparative thanatologists to over-intellectualise the CoD. Despite the care that they put into avoiding anthropomorphism when discussing animals’ thanatological responses, this over-intellectualisation reflects an anthropocentric stance. Rather than asking whether animals can develop anything that counts as a CoD, they ask whether they can develop a human-like CoD. We have argued that this is a biased and ecologically invalid way of approaching this issue. But over-intellectualisation is not the only way in which this implicit anthropocentrism manifests itself. In this subsection, we will argue that thanatologists not only assume that any understanding of death must take the human form, but also that there is only one possible way of emotionally reacting to death: the human way. We call this emotional anthropocentrism.

Brosnan and Vonk point out that there is a bias in the thanatological literature towards behaviours that are deemed ‘interesting,’ whereas observations of behaviours that are considered commonplace or boring are often left unpublished. They are worried that this could lead “to the perception of a greater frequency of ‘interesting’ or ‘positive’ results than is true in reality” (Brosnan and Vonk 2019, p. 99). This is once again the worry that we might be engaging in anthropomorphism. While this is a legitimate concern, we wish to highlight the anthropocentric bias implicit in the very delimitation of ‘interesting’ or ‘positive’ results. This bias is illustrated by the example that Brosnan and Vonk themselves use. They refer to the case of the orca nicknamed Tahlequah, who made the worldwide news after she was witnessed carrying her dead calf for 17 days and over 1000 miles (Cuthbert and Main 2018). As Brosnan and Vonk point out, Tahlequah’s behaviour could perfectly have been written up in a scientific report and published as a relevant finding. In contrast, if she’d been merely witnessed swimming away from her calf’s corpse and letting it sink to the bottom of the ocean, this would not have been deemed interesting enough to be worth publishing. However, what Brosnan and Vonk fail to note is that swimming away from a corpse is only uninteresting if we adopt an anthropocentric stance that favours human-like reactions to death.^12^ If what we’re looking for is evidence of a CoD, abandoning a corpse might be just as interesting as grieving behaviour.

The fact that the act of carrying a dead calf for 17 days is considered much more interesting or noteworthy than letting a corpse go points to a bias towards human-like emotional reactions to death. In Tahlequah’s behaviour we see a broken-hearted mother to whom we can all relate. As we saw in the previous section, humans attach a strong emotional significance to death.^13^ This not only leads comparative thanatologists to over-intellectualise the CoD, it also results in a fascination with those responses to death that resemble our own, something that ultimately blinds them to those that differ. For example, Anderson et al. describe the peaceful demise of an elderly female chimpanzee and explicitly associate the behaviours seen in the surrounding chimpanzees to human reactions that are typical of the grieving process, such as “anticipatory grief,” “attempted resuscitation,” “consolation,” “denial, feelings of anger towards the deceased,” and “leaving objects or places associated with the deceased untouched” (Anderson et al. 2010, p. R350). They consider that these similarities illustrate the “interest” of a comparative study of death and dying (Ibid.). However, it is only from an anthropocentric standpoint that we can consider animals’ reactions to death to become more interesting merely by being more human-like.

This emotional anthropocentrism has led to an excessive focus on primates, to the extent that the majority of thanatological reports concern species from this order.^14^ This does not necessarily mean that primates are more likely than other animals to react to death, but probably simply reflects the fact that they resemble humans in their anatomy, gestures, and social interactions. If what we’re looking for are human-like reactions to death, we are much more likely to find them in primates.^15^ Emotional anthropocentrism has also engendered an emphasis on affiliative behaviours towards corpses, to the extent that Reggente et al. characterise “death-related behaviour” as “a sub-category of epimeletic or nurturant behaviours” (Reggente et al. 2018, p. 1). This is because affiliation is an expression of attachment and, in the context of bereavement, a possible expression of grief. Accordingly, the thanatological literature is filled with reports of animals licking, grooming, embracing, protecting, and sleeping next to corpses (for reviews, see Anderson 2018; Goldenberg and Wittemyer 2020; Gonçalves and Biro 2018; Gonçalves and Carvalho 2019; Reggente et al. 2016, 2018), and the thanatological behaviour that has by far received the most attention is the prolonged carrying of infant corpses, seen most frequently in bereaved primate mothers (for reviews, see Das et al. 2019; Watson and Matsuzawa 2018).

Deceased infant carrying is an interesting behaviour to monitor, but we should be wary of focusing too much attention on it if what we’re interested in is animals’ CoD. Firstly, this behaviour has some peculiarities that make it problematic evidence of a CoD. There are usually very strong hormonal and emotional mechanisms underlying the prolonged carrying of infant corpses (Bercovitch 2020). If the mothers have a CoD, these other factors are likely to muddy the waters and make it difficult to discern whether they understand that their infant is dead. A CoD would allow them to process the change in their infant’s state, but these other hormonal and affective mechanisms could lead them to want to continue treating their baby *as if* she were alive. In fact, this is a behaviour *human* mothers are naturally drawn to when they have a stillbirth (Todorović 2016) and which is recommended for coping with parental grief (Kingdon et al. 2015). Secondly, giving this behaviour excessive attention perpetuates the idea that the only emotional reaction to death worth monitoring is grief. This could lead us to lose precious opportunities to unveil the prevalence of the CoD in nature.

We do not wish to call into question that some animals may grieve their dead. However, we believe that it is important to separate this from the question of whether animals can possess a CoD. Grief does not necessarily signal a CoD—what it signals is a strong emotional attachment to the dead individual. Accordingly, in social animals like chimpanzees, the deaths of infants trigger apparent mourning behaviour only from close relatives, whereas the deaths of older, more socially active group members generate emotional responses from many others, including non-kin (van Leeuwen et al. 2016). Furthermore, understanding that an individual has died does not necessarily imply grieving over her death or even *caring* about it, which makes the lack of reaction from non-kin to the deaths of infants compatible with a CoD. In fact, processing that someone has died can lead to all sorts of emotional responses, only one of which is grief. An animal may also be happy that another has died, if, for instance, this means a rise in the social hierarchy. Or she may feel excitement or hunger, if she perceives the corpse as a source of food. Or she may feel a self-centred form of sadness, if she has lost her favourite back-scratcher. Or she may simply not care, if she has no particular connection to the dead individual. All of these reactions^16^ are compatible with processing that the dead individual has lost the cluster of functions characteristic of living beings of her sort and that this change in her state is irreversible. In turn, these emotional responses can lead to behaviours such as cannibalism or necrophilia, which are far removed from what we expect from an anthropocentric perspective. In order to be able to incorporate these different emotional and behavioural responses, the question of animals’ CoD should be treated independently of the question of animal grief.

## Beyond anthropocentrism: the prevalence of the CoD in nature

In the previous section, we have argued for the importance of not over-intellectualising the CoD and of treating it as a separate question to that of animal grief. This ultimately means abandoning an anthropocentric perspective on death. Having laid down this groundwork, in this section we will defend the main thesis we want to put forward: if we relinquish intellectual and emotional anthropocentrism about death, we can expect the CoD to be both relatively easy to acquire and fairly widespread in nature. We will defend this thesis by, firstly, establishing the necessary requirements for a CoD to emerge, and secondly, illustrating the multiple ways in which animals in nature can learn about death.

### Prerequisites for a CoD

The reader may have noticed that we have added two qualifiers to our main thesis: the CoD is *relatively* easy to acquire and *fairly* widespread in nature. These two qualifiers are meant to capture that the CoD cannot be acquired by *any* animal. There are, in fact, three threshold conditions that have to be met for an animal to be a potential candidate for possessing a CoD: we call these cognition, experience, and emotion.

1. cognition: The CoD is more than the capacity to perceptually discriminate dead bodies; it requires the ability to process non-functionality and irreversibility.
2. experience: The CoD is not innate.^17^ Instead, it depends on the accumulation of experiences with death that can allow for learning to occur.
3. emotion: Developing a CoD requires attending to the dead and the dying, in order to learn about irreversible non-functionality. Some kind of emotional mechanism is needed to provide the adequate motivation. We intend the phrase ‘emotional mechanism’ in a broad sense, to encompass affects, moods, and behavioural drives with emotional components. Thus, we need the animal to be capable of things such as love, curiosity, boredom, excitement, and so on, which can move her to attend to others’ death.

These three components (experience, cognition, emotion) are all necessary to a certain extent in order for the CoD to emerge. An animal who lived in isolation would never have experiences with death and could therefore never learn about it. An animal who were incapable of anything but very rigid responses to perceptual cues could never acquire a CoD. And lastly, attention strongly depends on emotions (Huntsinger 2013; Machado et al. 2011; Zikopoulos and Barbas 2012), so an animal who lacked the latter would not attend to death. While these three components must all be present to a *certain* extent, the high presence of one can compensate for the relative absence of another. Thus, for instance, a particularly intelligent creature might not need more than a few experiences with death in order to learn about it. In contrast, a creature with hundreds of experiences with death might not need to be too bright in order to develop a minimal CoD. And an individual who loses someone that she is highly attached to might be so motivated to stay close to the corpse and pay attention to it that she can come to grasp its irreversible non-functionality even if she has had no other experiences with death. These three threshold conditions, as we will see, allow us to make predictions regarding where to expect a CoD to emerge in nature.

Some comparative thanatologists have postulated sociality as a key factor in the emergence of a CoD (Appleby et al. 2013; Bearzi et al. 2018; Bercovitch 2020; Gonçalves and Biro 2018). For instance, Bearzi et al. (2018) argue that sociality facilitates the emergence of strong social attachments that are unlikely to simply vanish with death. These attachments, in turn, lead animals to pay attention to the corpses of those they are bonded with, and thus give them the chance to learn about death. This point is echoed by Bercovitch, who notes that the comparative thanatology literature is exclusively composed of reports concerning social animals, and that there are no descriptions of reactions to the dead in solitary species, such as koalas or moose (Bercovitch 2020, p. 23). It has also been argued that the fission–fusion dynamics that characterise some social species could be crucial in the development of a CoD, insofar as these societies require the individuals to be constantly monitoring and updating the status of other individuals in the group (Goldenberg and Wittemyer 2020; Piel and Stewart 2015).

While sociality could be a facilitator in some cases, we do not think that it should be considered a prerequisite for developing a CoD. If sociality is a reliable indicator of species in which the CoD can emerge, it is only insofar as it is correlated with high levels of cognition, emotion, and experience. As these authors note, some social animals develop strong social bonds (= emotion). They also tend to live in social groups, and mortality rates in nature are high (= experience). And lastly, sociality has been proposed as a factor in the evolution of certain kinds of intelligence, and high cognitive ability in turn usually requires a slow development, which favours sociality (= cognition). However, sociality on its own is not a prerequisite for the emergence of a CoD, because not all social animals have high levels of cognition, emotion, and experience. While in mammals it has been argued that group size could be a predictor of brain size (Dunbar 1998), in some social insects the opposite effect has been found, with sociality having an inverse correlation to cognitive complexity at the individual level (O’Donnell et al. 2015). Social insects likely also have too little cognitive complexity to ever be capable of developing a CoD, instead responding to the dead in largely stereotypical ways that are dependent on chemical cues (Sun et al. 2018; Sun and Zhou 2013). Moreover, some species are non-social but still have high enough levels of cognition, emotion, and experience to be likely candidates for possessing a CoD. In particular, our model predicts that large predators, which are for the most part non-social, are strong candidates for a CoD, even though they are largely ignored in the thanatological literature. While, as Bercovitch (2020) points out, the available thanatological reports concern only social species, all this does is reflect the bias, explained in the previous section, towards reporting on behaviours that are deemed ‘interesting,’ i.e., human-like.

The type of experience an animal needs to learn about death is clear. But what type of cognitive and emotional mechanisms do animals require in order to develop a CoD? We need the animal to attend specifically to the functionality of the living, and to be able to contrast it with the irreversible non-functionality of the dead. We have strong reasons for thinking that this capacity will be widespread in nature. Firstly, vertebrates tend to be very good at distinguishing biological from non-biological motion (Johnson 2006; Troje and Westhoff 2006; Vallortigara and Regolin 2006) and can easily categorise entities as animate or inanimate, having for instance certain expectations about how inanimate objects behave (Takagi et al. 2016) and being wary of those that violate them (Greggor et al. 2018). As Gonçalves and Carvalho (2019) note, being able to tell these two types of entities apart is crucial for survival in the wild. In contrast to inanimate entities, animate beings act in a self-propelled and goal-directed manner without the influence of an external force. This makes their movement more difficult to predict and, coupled with the prevalence of violence in nature, much more important to monitor. For this reason, animals are primed towards paying attention to any signs of animacy and goal-directed behaviour in their surroundings (Call et al. 2004; Marshall-Pescini et al. 2014; Phillips et al. 2009). This is all the more important given the widespread distribution in nature of concealment methods, such as mimicry or camouflage (Gonçalves and Carvalho 2019, p. 1515).

Not only are animals biased towards paying attention to animacy, there is also a widespread capacity to learn patterns and develop implicit cross-modal expectations regarding how the animate entities in one’s surroundings *typically* behave (Adachi et al. 2007; Proops et al. 2009; Takagi et al. 2019). This also has high survival value, since it can allow an animal to predict the behaviour of friends and foe, as well as detect anomalous behaviour. Any behaviour that departs from the norm potentially signals a threat (e.g. a predator, a parasite, or a poisonous food source) or an opportunity (e.g. a prey animal that is easier to catch or an alpha male that could be overthrown). As we discussed in Sect. 2, the minimal CoD does not require an explicit notion of ‘animacy’ or ‘life,’ but simply for the animal to have developed implicit expectations regarding how beings of a certain kind typically behave. Given its high survival value, we can expect natural selection to have favoured the development of this capacity in many species. Any animal who can develop expectations regarding the typical behaviour of other animals will likely be able to sense the total lack of behaviour characteristic of corpses.

These considerations support the idea that the ability to process non-functionality will be widespread in nature. But the minimal CoD is not just composed of non-functionality, but of irreversibility too. There could be the temptation to think that processing irreversibility is much harder than processing non-functionality, given that irreversibility seems to incorporate a *temporal* component (i.e., the idea that the dead individual will not exhibit living functions *anymore*). This has led some to speculate that the CoD requires a capacity to mentally time-travel or to reason about the future (e.g. Brosnan and Vonk, p. 81). If this were true, we would perhaps expect fewer species to be capable of developing a CoD [although mental time-travel is likely more widespread than is often assumed (Zentall 2013)]. However, we believe that irreversibility can be understood in less demanding terms, as an ability that is independent of temporal reasoning. The idea is that processing irreversibility means *recategorising* an animal from one that is expected to exhibit the functions typical of her kind to one that is *not* expected to exhibit these functions. This is different from, for instance, how an animal might process the non-functionality of an asleep individual. An animal who is asleep is not exhibiting many of the functions of living individuals, but she is still an animal from whom one can expect these functions (i.e., she is *reversibly* non-functional). Processing irreversibility doesn’t require an ability to reason about the future, but simply an ability to break away from a particular expectation and generate a new one. Any animal who can learn will likely be able to alter her expectations subject to her experience. Thus, we can expect many species to fulfil the minimal cognitive requirements to learn about death. In the following subsection, we specify further reasons for thinking that the CoD will be widespread in nature.

### The multiple ways in which animals can learn about death

When considering the different ways in which animals can learn about death, something crucial that is not sufficiently stressed in the thanatological literature is that a corpse is *very* different from a live individual. Scientists discussing deceased infant carrying sometimes speculate that the mothers may not have correctly processed the change of state in their infant (Biro et al. 2010; Sugiyama et al. 2009; Watson and Matsuzawa 2018). This becomes implausible if one considers (a) that mothers will typically have seen and interacted with plenty of live infants and (b) the multi-modal cues that accompany death. A corpse is not like an asleep or unconscious individual. Even a fresh corpse *looks* different in its absolute stillness; it *feels* different in its coldness, limpness, and total absence of responsive feedback; it also *sounds* and most probably *smells* different to animals with an acute sense of smell. Mothers who carry their deceased infants for days, or even weeks, are bound to notice at least *some* of these differences. This is reflected in their adoption of carrying techniques that are never used on live infants (Biro et al. 2010; Carter et al. 2020; Das et al. 2019; Fashing et al. 2011).

The multi-modality of a corpse’s non-functionality appears to be exploited by animals in their inspection of dead bodies. Primates, for instance, have often been witnessed carefully and insistently looking at the faces of corpses (Boesch and Boesch-Achermann 2000; Cronin et al. 2011; De Marco et al. 2018, 2020; Fashing et al. 2011; Pruetz et al. 2017; Yang et al. 2016). Regardless of whether this behaviour is curiosity-driven or the result of a natural tendency to fixate on faces, this body part offers visual cues that have a communicative function, which presupposes that conspecifics can interpret them—and consequently makes it likely that they will sense their total absence. Tactile investigation is also important. Watts (2020) recounts of a female gorilla who, after several hesitant attempts to move away from her infant’s corpse, pulled gently on its leg a few times before abandoning it altogether. Tactile interactions also include aggressive or sexual behaviour towards corpses. Stewart et al. (2012) describe a group of chimpanzees rough-handling a conspecific’s corpse and dragging it over 60 metres. Bearzi et al (2018) note how cetaceans will often engage in necrophilia, possibly as a show of dominance or because the stress of the situation generates sexual arousal. Similarly to the inspection of faces, these behaviours may not be done with the *intention* of investigating the corpse’s non-functionality, but they will offer clear information on the corpse’s lack of responsiveness. Olfaction is also used in the examination of corpses. Trapanese et al. (2020) report on a male capuchin monkey who inspected the anus of a stillborn infant and, immediately after, that of a live infant, possibly as a way of comparing olfactory cues. For elephants, smell may be the most salient characteristic of corpses, since they appear able to distinguish conspecific from heterospecific bones using olfaction and have also been witnessed smelling the ground where a corpse has decayed (Goldenberg and Wittemyer 2020).

The multi-modal character of a corpse’s non-functionality offers many chances for an animal’s expectations to be broken and for her to process that the individual in question is not responding and behaving in the way beings of her kind typically do. But, additionally, corpses come with their own functionality, which is also multi-modal (Gonçalves and Biro 2018; Gonçalves and Carvalho 2019). They start to smell strongly and characteristically, they become bloated, putrefy, and get infested with maggots, or they mummify and slowly dry up and disintegrate. In certain climates, these changes can happen quickly. Teleki (1973), for instance, reports that in the tropical forests of equatorial Africa corpses start to show signs of putrefaction merely 8 hours after death. This may allow the surrounding animals to have a clear sense that this is the same individual who was alive a few hours ago. But even if these changes happen more slowly, animals will typically spend their whole lives in the same environment, and so corpses will roughly follow the same patterns of change. This can allow the animals to learn about the irreversible nature of death, insofar as once these signals appear, the individual never again shows signs of life.

Thanatologists not only tend to pay little attention to the multi-modality and functionality of death, they are also, as we saw in the previous section, fixated on intra-specific relations and affiliative or otherwise noteworthy behaviours towards corpses. But death is everywhere in nature. Animals typically share their environment with many other species, with regards to whom they will likely also have formed implicit expectations of how they behave. In addition, mortality rates are very high in the wild. It is generally difficult for an animal to make it to maturity, given the high prevalence of infant mortality, even among long-living animals with slow developments [e.g. half of Mahale chimpanzees die before weaning (Nishida et al. 2003); cub mortality amongst lions may be as high as 67% (Schaller 1976)]. But even animals who reach adulthood face a constant threat of death, due to the high occurrence of predation, disease, parasitism, accidents, natural disasters, lack of resources, intra-specific violence, and human-related causes. Thus, animals will encounter death in many different forms throughout their lives. Many, or perhaps most, of these deaths will occur in animals of other species towards which the witnessing individuals have no emotional attachment, but still the number of deaths witnessed (= high experience) could compensate the relative lack of interest (= low emotion). Thus, they could contribute to the animals’ development of a CoD, even if they might not generate any ‘noteworthy’ behavioural responses other than simply ignoring the corpse.

The focus on affiliative behaviours and the search for grief has also led to the disregard of deaths that are the result of purposeful killing. This is unfortunate, given that they potentially represent a fertile ground for the CoD to emerge, both for the perpetrator (insofar as being the cause of the event likely makes the characteristics of death more salient) and for members of the social group of the victim [violent episodes tend to generate interest or alertness, and they are also accompanied by strong sensory cues of death in the form of wounds (Gonçalves and Carvalho 2019)]. In some cases, these events could provide suggestive evidence of a CoD. Kaburu et al. (2013), for instance, recount the coalitional killing of the alpha male in a chimpanzee community. The perpetrators spent more than two hours attacking the victim before he died. The authors write: “The escalation of violence beyond the necessary for AL to defeat PM remains difficult to explain. The attackers, in particular DE, seemed intent on ensuring PM’s death” (Kaburu et al. 2013, p. 794). Another potential behaviour of interest is infanticide, which is widespread in the animal kingdom and a leading cause of infant mortality in some species. For example, Brown et al. (2020) found that 21% of infant deaths in a population of hyenas were due to infanticide; in chimpanzees this figure can go up to 63% (Lowe et al. 2020). Discussions on this behaviour tend to revolve around its ultimate function, with comparatively little analysis of what motivates infanticide at the proximate level. Although a handful of papers discuss infanticide and other intra-specific killings in relation to animals’ understanding of death (Bearzi et al. 2018; Fedurek et al. 2020; Gonçalves and Carvalho 2019; Trapanese et al. 2020; Anderson 2018), this remains an under-explored topic.

It is even rarer to come across a discussion of *inter-specific* killings in the comparative thanatology literature, and when this appears it is always as a side-note rather than the main focus (e.g. Anderson 2018; Watts 2020). A big gap in the literature is thus the topic of predation. Large predators tend to be cognitively complex and they have both a tendency and an incentive to track non-functionality in prey, in order to target those animals that are old, young, sick, disabled, injured, or show any other signs of partial non-functionality. This is a well-known ecological phenomenon called the ‘sanitation effect’ (Crisler 1956; Leopold 1987; Lingle and Wilson 2001), and it depends not just on the relative weakness of those individuals, but also on the predators’ sensitivity to extremely subtle differences in behaviour (Krumm et al. 2010). Moreover, although predators have no emotional attachment to their prey, they have a strong interest in hunting them down. Due to the evolutionary arms race between predators and their prey, they often have low success rates [e.g. lions have only a 13% chance of catching a topi and a 26% chance in the case of gazelles (Schaller 1976, p. 389)], which gives them an incentive to attend to their prey’s death. This incentive is also provided by the fact that predators are at considerable physical risk from apparently dead prey suddenly reviving and lashing out.^18^ Lastly, predators who make it to maturity can accumulate hundreds of experiences with death. The same goes for prey animals who live in social groups under the constant threat of predation. They also have a strong incentive to attend to predatory behaviour, and are often intimately aware of the potential of each predator, to the extent that they adapt their flight distance to the danger that each species represents (Ibid., p. 387). If they are lucky and survive long enough, they will also witness many of their fellows succumbing to predators. It is thus surprising that the literature all but ignores this topic.

A further reason for thinking that predation could be a hotspot for the emergence of the CoD in nature has to do with a common defence mechanism found in prey, one that has appeared independently on multiple occasions throughout evolutionary history. We are referring to thanatosis, or death feigning. Some species, in situations of high stress, enter a state of stillness that reduces their probability of being preyed upon. This can be found in a wide variety of organisms, from spiders (Hansen et al. 2008) to human beings (Kalaf et al. 2017), generally as a stress-mediated response to dangerous situations where running away or hiding is not an option. Humphreys and Ruxton (2018) have argued that the term ‘tonic immobility’ is preferable to ‘thanatosis,’ because the fact that a paralysed animal seems dead to our human eyes doesn’t mean that the reason why evolution has favoured it is because she appears dead to the eyes of predators. We agree that most of the evolutionary success of these behaviours probably comes from the elimination of certain perceptual stimuli that attract the attention of predators (Tremoulet and Feldman 2006), without any need from them to categorize the performer as a corpse. Additionally, an important proportion of what might look like thanatosis is not much different from a paralysis. In insects, for instance, an analysis beyond bodily movement or posture reveals the shallowness of this behaviour (e.g. heart rate and breathing speed up during this state Nishino 2004; Rogers and Simpson 2014). We believe that the term ‘tonic immobility’ should indeed be preferred in these cases.

However, in some cases (especially, but not exclusively, in mammals), this defence mechanism involves not only a removal of (some of) the perceptual characteristics of living beings, but also a series of mechanisms that go further than what ‘tonic immobility,’ as a term, describes. In some mammals, heart rate slows down, breathing is reduced, body temperature drops, there is salivation, urination, defecation, and even the bodily and facial expression may imitate a corpse. In the most notorious cases, this death imitation goes even further: in Virginia opossums, the tongue turns blue and the anal glands simulate the smell of rot (Gabrielsen and Smith 1985), and grass snakes can secrete blood from their mouth and nose while ‘playing dead’ (Bartlett 1920). We believe that the term thanatosis is perfectly apt in these cases, since there is a clear death imitation. Obviously, this does not mean that the performer has a CoD. In the same way that the mimicry of a ghost insect allows it to look and behave like a leaf rocked by the wind (Bian et al. 2016) without requiring a concept of leaf or wind, the thanatosis performer does not need to have a CoD to be able to carry it out. However, the fact that this behaviour was *selected* points to the likely prevalence of a CoD amongst *predators*.

It has been proposed that thanatosis would be effective against those predators that try to catch as much prey as possible in a limited time. Playing dead would encourage the movement of the predator to the next prey, thus giving the performer a chance to escape (Humphreys and Ruxton 2018). Experimental evidence also suggests that thanatosis is a successful strategy if the predator has previously learned that dead prey is unpalatable, which has been taken to mean that “predator cognition is likely to be a key selective pressure driving the evolution of thanatosis” (Skelhorn 2018, p. R1122). It has also been argued that thanatosis would be more effective against generalist predators, which don’t specialise on a single type of prey and cannot easily adapt to these kinds of tricks (Gregory et al. 2007; Humphreys and Ruxton 2018). Regardless of the concrete evolutionary story, if thanatosis evolved, it’s because there is an advantage, not just to keeping still, but specifically to appearing dead. In cases like the Virginia opossum and the grass snake’s, the behaviour is highly complex and comprises a heterogeneous cluster of traits whose only commonality is that they are characteristics of death. Thus, it is difficult to see what other selective pressure could have shaped it if not the CoD of the deceived predators. We believe that this is not a difficult conclusion to accept once we leave aside our anthropocentric biases and acknowledge that the CoD, far from being a uniquely human feat, is likely prevalent in nature.

## Conclusion

In this paper, we have argued against the assumption, common amongst comparative thanatologists, that a CoD is difficult to acquire and very rare once we move past the human species. We have shown that this assumption stems from two forms of anthropocentrism: an intellectual anthropocentrism that leads scientists to over-intellectualise what understanding death amounts to, and an emotional anthropocentrism that generates an excessive focus on human-like emotional reactions to death. We have illustrated how, once we identify and remove these biases, it becomes clear that the cognitive requirements for a CoD are quite widespread in nature, and that there are multiple pathways and opportunities for animals in the wild to learn about death. If our arguments are correct, the CoD is likely much more common in nature than is usually presupposed.

Two final objections could be raised at this point. The first one would consist of saying that the conclusion we have reached is actually not that surprising because we have set a very low bar for the attribution of a CoD. However, it has been precisely our aim in this paper to argue that the bar *should* be set very low, and that any attempts to raise it stem from unwarranted anthropocentric biases. The second objection might consist of a defence of the anthropocentric outlook, by saying that the main reason why one would want to deny a CoD to other species has to do with the strong significance that death and mortality have in our human lives and practices. This sort of significance, one could argue, cannot be captured from a naturalistic point of view, but necessarily requires an anthropocentric, humanistic perspective. We could thus be accused of having changed the topic from a discussion of a concept that fundamentally defines our lives to an investigation of one that is devoid of all human meaning. We acknowledge this, but want to stress that our paper is a defence of the need for comparative thanatologists to ‘change the topic’ in this sense. We believe that it is perfectly legitimate to deny that animals can have *our* CoD, but from the perspective of comparative thanatology the interesting question, we believe, is whether they can have anything that counts as a CoD at all. Our aim in this paper has been to argue that there are strong reasons for thinking that many animals likely do have something that counts as a CoD. However, this is compatible with thinking that the human perspective on death is likely unique in many regards.

## Footnotes

1 Throughout this paper, we use the term ‘animal’ to refer to nonhuman animals.

2 We are using this term in a loose sense, to refer to scientists who have dealt with animals’ reactions to the dead and the dying. In a majority of cases, this is not their primary topic of study, since most thanatological reports consist of opportunistic observations in the wild.

3 These two types of responses to death (stereotypical vs cognitive) are not mutually exclusive in a single species. In humans, for instance, we find both sophisticated cognitive and emotional responses to death and an automatic activation of threat-management responses in reaction to the chemical cues of corpses (Wisman and Shrira 2015).

4 We take anthropocentrism to be something distinct from anthropomorphism. We are using the term ‘anthropomorphism’ to refer to the incorrect attribution of human-like qualities to animals, whereas we understand anthropocentrism to refer to a bias by which human experience is taken as the gold standard and the lens through which all animal behaviour ought to be interpreted. Naturally, some anthropocentrism is inevitable—we can’t, after all, transcend our own human nature, and so it would be futile to attempt to eliminate all bias towards our own experiences. However, we believe that this bias can be corrected to an important degree by bringing attention to it and making the conscious effort of being open-minded to the alternative ways in which animals might experience the world. Our aim in this paper is to show how, coupling this sort of open-mindedness with biological and ecological considerations, we can reach the conclusion that the CoD is probably fairly widespread in nature.

5 One could question whether humans can ever grasp other ways of understanding, a worry expressed in Wittgenstein’s assertion that “[i]f a lion could talk, we wouldn’t be able to understand it” (2009, p. 227). We are not as pessimistic as Wittgenstein in this regard. We believe that there are certainly limitations in our ability to access how animals represent the world, but that longitudinal observational studies, together with experiments performed under controlled conditions, can get us quite far in delineating animals’ perspectives on the world. For suggestions on how to experimentally test for animals’ CoD, see Allen and Hauser (1991), Gonçalves and Biro (2018), and Monsó (2019).

6 In this paper, we will refrain from entering into the philosophical debate on concept possession, since this topic is not considered in the thanatological literature and discussing it would lead us too far astray. Accordingly, we follow Monsó (2019) in using the term ‘concept’ somewhat loosely and interchangeably with the terms ‘notion’ and ‘understanding,’ to signify an ability to classify individuals as having a certain property, with relative independence of the perceptual cues that signal that property and a grasp of the intensional features of that property. This definition is sufficient for our purposes, since it captures the loose sense in which the term ‘concept’ is used in comparative thanatology. For a discussion of how this connects to philosophical debates on concept possession, see Monsó (2019, Sect. 2).

7 One cannot speak of a single, homogeneous CoD among humans, since there is significant variability in how different cultures conceptualise death and dying (Gire 2014). Comparative thanatologists tend to have *one of* the human CoDs in mind, namely, the one that corresponds to the scientific understanding of death.

8 A reviewer suggests the following counterexample: “Consider an individual [let’s call him Tom] who is permanently paralysed (e.g. by a stroke). A subject, S, could deem that clauses (a)–(c) are satisfied by [Tom] without S applying or having a CoD.” In responding to this counterexample, we need to first distinguish the hypothetical case in which S lacks a CoD from the case in which she judges Tom to satisfy clauses (a)–(c) but does not consider him dead. If S lacks a CoD, then this counterexample is not so problematic. We would simply say that in judging ToM to satisfy clauses (a)–(c) she is beginning to develop a CoD. After all, Monsó’s definition does not require full reliability in the classification of individuals as dead, and presumably the concept that S is developing would allow her to correctly discriminate actual dead individuals in the future. If, however, S has a CoD but is just not applying it in this particular instance, then the counterexample becomes trickier to deal with, but not impossible. We might say, first of all, that S is mistaken in her attribution of clause (b) to Tom, given that he’s still exhibiting the minimal cluster of living functions (e.g. cardiopulmonar activity). However, the reviewer suggests, “what if [Tom] can’t actually do those things for himself and needs to be on a heart and lung machine?” In this case, we would say that Tom’s constitutes the sort of borderline case regarding which there are significant debates in bioethics (see DeGrazia 2017), so that it’s not at all clear whether S would be right in believing that Tom is not dead. But importantly, within these debates there is widespread agreement that death is the irreversible cessation of the functions characteristic of living human beings: the disagreement revolves around the precise delineation of these functions (Ibid.). The answer to whether or not S is mistaken in believing that Tom is not dead will therefore depend on whether we are willing to grant that functions sustained by machines external to the organism are part of the living functions of that organism. We believe that the vagueness of the phrase “the cluster of functions characteristic of living beings” in Monsó’s definition allows for the incorporation of different understandings of these sorts of borderline cases. However, those readers who are uncomfortable with any attempt to define concepts using necessary and sufficient conditions can instead think of this definition as one specifying (defeasible) evidence for the attribution of a CoD. Paraphrasing Allen (1999, p. 37), the satisfaction of these conditions provides good grounds for the attribution of a CoD, even if one does not wish to view them as necessary and sufficient for the possession of a CoD.

9 The minimal CoD is a theoretical construct; it establishes the bare minimum that an animal needs to process in order to be credited with an understanding of death. But it is not necessarily the starting point in an animal’s learning about death; it is possible that in nature the CoD emerges in more than its minimal form. In this paper we do not wish to take a stand on the exact form that the natural CoD will take, but rather argue that the minimal components of a CoD are easy to acquire and therefore likely prevalent among wild animals.

10 According to terror management theory, a strong drive behind human behaviour is our need to counter the terror evoked by the knowledge of our inevitable demise, which leads us to incorporate attitudes and behaviours, such as religiosity, that give us the sense that our lives have enduring meaning and thus minimise this terror. This is connected to Heidegger’s (1996) remark that humans tend to live inauthentic lives, since we fear death and treat it as something that is expected rather than something that is anticipated, and thus owned. In this regard, one might question the extent to which the CoD is actually present in human lives, since we seem to have many psychological mechanisms for ignoring our own mortality. However, we take it as a given that all cognitively abled human adults have a CoD, regardless of how much we avoid thinking about death during the course of our lives, given that we all have a notion of what it means to be dead and can discriminate dead individuals with high reliability.

11 We use this term, following Henrich et al. (2010), to refer to Western, educated, industrialised, rich, and democratic human societies.

12 We should note that this anthropocentric stance is also ethnocentric, insofar as within some human cultures death is not seen as a tragedy (Gire 2014).

13 Naturally we, as authors of this paper, also attach a strong emotional significance to death, so one could question our ability to counter this emotional anthropocentrism. While it is indeed challenging and there are of course limitations in our ability to transcend our own CoD, we think that we can go a long way by coupling what we know about the needs and psychology of different species and how they interact with their environment with a conscious effort to bracket our own perspective on mortality.

14 Due to this anthropocentric bias in the literature, many of the empirical findings we refer to in this paper are on nonhuman primates.

15 The excessive focus on primates is also the result of intellectual anthropocentrism. Insofar as scientists believe the CoD to be a very demanding cognitive feat, they are more likely to look for it in species that are considered more cognitively complex.

16 One could worry that the emotional reactions described in this paragraph are anthropocentric or anthropomorphic. This is a legitimate concern. However, we are not asserting that these necessarily represent actual emotions that animals have. Rather, our aim is to illustrate that the CoD is in principle compatible with a myriad of emotional reactions besides grief.

17 Some species, such as crows, are equipped with adaptations that prime them towards paying attention to cues of death (Swift et al. 2020; Swift and Marzluff 2015), and perhaps make it easy for them to develop a CoD. But even in cases like this, without *some* experience with death the animal will not be able to make use of these adaptations, and the CoD will remain elusive.

18 We thank an anonymous reviewer for pointing this out to us.
